# From system to organ to cell: oxygenation and perfusion measurement in anesthesia and critical care

**DOI:** 10.1007/s10877-012-9350-4

**Published:** 2012-03-22

**Authors:** Patrick Schober, Lothar A. Schwarte

**Affiliations:** Department of Anaesthesiology, VU University Medical Center, De Boelelaan 1117, 1007 MB Amsterdam, The Netherlands

**Keywords:** Oxygen, Oxygenation, Macrocirculation, Microcirculation, Tissue, Cell

## Abstract

Maintenance or restoration of adequate tissue oxygenation is a main goal of anesthesiologic and intensive care patient management. Pathophysiological disturbances which interfere with aerobic metabolism may occur at any stage in the oxygen cascade from atmospheric gas to the mitochondria, and there is no single monitoring modality that allows comprehensive determination of “the oxygenation”. To facilitate early detection of tissue hypoxia (or hyperoxia) and to allow a goal directed therapy targeted at the underlying problem, the anesthesiologist and intensive care physician require a thorough understanding of the numerous determinants that influence cellular oxygenation. This article reviews the basic physiology of oxygen uptake and delivery to tissues as well as the options to monitor determinants of oxygenation at different stages from the alveolus to the cell.

## Introduction

Oxygen uptake from atmospheric gas in the lungs and transport to tissues are pivotal steps to allow oxidative phosphorylation in mitochondria. Adenosine triphosphate (ATP) formed during this final step of cellular respiration serves as the fuel to maintain cellular homeostasis and metabolism. The rate at which oxygen deprivation results in depletion of ATP stores and subsequent cell damage depends on the organ’s metabolic oxygen demand as well as its capability to derive ATP from anaerobic metabolism. Anaerobic ATP production, however, is insufficient to match metabolic needs and therefore only delays but cannot prevent cellular injury. Therefore, whenever cellular oxygen demand exceeds oxygen supply, organ dysfunction and even irreversible damage may readily occur depending on the degree and duration of oxygen deprivation [1]. Hence, maintenance or restoration of adequate tissue oxygenation is a main goal of anesthesiologic and intensive care patient management. However, in recent years also the risk of hyperoxemia to cause organ damage by exerting oxidative stress has come into the focus of clinical interest [2]. Therefore, monitoring of systemic and regional oxygenation is not only important to prevent tissue hypoxia, but may in the future also play an important role to detect hyperoxemia.

Pathophysiological disturbances which interfere with aerobic metabolism may occur at any stage in the oxygen cascade from atmospheric gas to the mitochondria, and there is no single monitoring modality that allows comprehensive determination of “the oxygenation”. To facilitate early detection of tissue hypoxia (of hyperoxia) and to allow a goal directed therapy targeted at the underlying problem, the anesthesiologist and intensive care physician require a thorough understanding of the numerous determinants that influence cellular oxygenation. This article reviews the basic physiology of oxygen uptake and delivery to tissues as well as the options to monitor determinants of oxygenation at different stages from the alveolus to the cell. While each of these monitoring techniques has been reviewed in detail elsewhere, the aim of this review is to provide a broad overview over possibilities, indications and limitations of available techniques.

## Physiology of oxygen uptake and transport

Gas diffusion from alveolus to capillary is driven by the gradient in the partial pressure of oxygen (pO_2_) between alveolus (p_A_O_2_) and the surrounding capillaries. It is proportional to the total area of the alveolocapillary membrane available for diffusion in the lung and inversely proportional to the diffusion distance across the membrane. The area as well as the diffusion distance can be altered by pulmonary or non-pulmonary disease (e.g., lung edema due to congestive heart failure), and therapy is primarily directed at the underlying cause. In contrast, the alveolar p_A_O_2_ can easily and quickly be manipulated therapeutically by altering the inspiratory oxygen fraction (F_i_O_2_), and therefore plays an important role in the immediate symptomatic treatment of impaired pulmonary oxygen uptake. The relationship between p_A_O_2_ and F_i_O_2_ can be described by the alveolar gas equation [3]:

$$ {\text{p}}_{\text{A}} {\text{O}}_{2} = {\text{F}}_{\text{i}} {\text{O}}_{ 2} \cdot ({\text{P}}_{\text{B}} - {\text{P}}_{{{\text{H}}_{ 2} {\text{O}}}} ) - \frac{{{\text{p}}_{\text{a}} {\text{CO}}_{2} }}{\text{RQ}}, $$

where P_B_ is barometric pressure (760 mmHg at sea level), ${\text{P}}_{{{\text{H}}_{ 2} {\text{O}}}}$ is the saturated water vapour pressure at the patient’s body temperature (47 mmHg at 37 °C), p_a_CO_2_ is the arterial partial pressure of carbon dioxide, and RQ is the respiratory quotient, i.e., the ratio between CO_2_ production and oxygen consumption (normally about 0.8). ${\text{P}}_{{{\text{H}}_{ 2} {\text{O}}}}$ and RQ are relatively constant and barometric pressure becomes a concern only when treating patients at higher altitudes [4]. Carbon dioxide partial pressure only has small influences in its physiologic range, but note that marked hypercapnia will inevitably lead to hypoxia in patient’s breathing room air when p_a_CO_2_ exceeds about 80 mmHg [5].

While oxygen diffusion across the alveolus normally results in an equilibration between alveolar and end-capillary pO_2_, arterial pO_2_ is physiologically lower due to small anatomic right–left shunts (bronchial and Thebesian veins) as well as physiologic ventilation/perfusion mismatch in the lung (venous admixture in areas with low ventilation/perfusion quotient). Pathologically increased venous admixture due to alveolar collapse, e.g., in patients with Acute Lung Injury (ALI) or Acute Respiratory Distress Syndrome (ARDS), increase the gap between alveolar and arterial pO_2_ (AaDO_2_) and may readily lead to arterial hypoxemia. Likewise, pulmonary edema can increase AaDO_2_ and cause hypoxemia [6].

In blood, oxygen is mainly bound to hemoglobin and only a small amount is dissolved in plasma. One gram of hemoglobin carries about 1.34–1.39 mL of oxygen and the dissolved amount of oxygen is proportional to its partial pressure, with the solubility coefficient for oxygen in plasma (0.0031 when p_a_O_2_ is expressed in mmHg) being the coefficient of proportionality. These properties allow calculation of arterial blood oxygen content (C_a_O_2_):

$$ {\text{C}}_{\text{a}} {\text{O}}_{ 2} = {\text{S}}_{\text{a}} {\text{O}}_{ 2} \cdot [{\text{Hb}}] \cdot 1.34 + 0.0031 \cdot {\text{p}}_{\text{a}} {\text{O}}_{2} , $$

Since the quantity of dissolved oxygen is normally negligible, arterial oxygen saturation (S_a_O_2_, expressed as a ratio of 1) and hemoglobin concentration ([Hb], in g/dL) are the two major determinants of arterial oxygen content. The relationship between oxygen saturation and p_a_O_2_ is described by the oxygen-hemoglobin-dissociation curve. Assuming physiologic values of [Hb] and SO_2_, normal oxygen content of arterial blood is about 180–200 mL oxygen per L blood [7]. Systemic oxygen delivery (DO_2_) to the organs, in turn, can be calculated as the product of cardiac output (CO) and arterial oxygen content (C_a_O_2_) [8]:

$$ {\text{DO}}_{2} = {\text{CO}} \cdot {\text{C}}_{\text{a}} {\text{O}}_{2} $$

The relative amount of the cardiac output delivered to an organ determines oxygen delivery to that particular organ. While different organs receive a relatively predictable blood flow under physiologic conditions depending on their metabolic demand, shock states are often associated with a redistribution of blood flow due to systemic vasoconstriction (e.g., hypovolemic shock) or systemic vasodilation (e.g., in septic shock). Other factors such as shunting on microcirculatory level, regional vasodilation or constriction due to local metabolic factors, stenosis of vessels or elevations of tissue pressure counteracting adequate perfusion can all result in an inhomogeneous perfusion and oxygenation of tissues. Therefore, macrocirculatory variables indicating adequate global oxygen delivery do not necessarily predict adequate perfusion and oxygenation of individual tissues. Table 1 summarizes the main determinants of tissue oxygenation, and monitoring of key determinants of oxygenation will be reviewed in the following section.

**Table 1:** Determinants of oxygen delivery to tissues and tissue oxygenation

Arterial oxygen content, defined by [Hb], SaO_2_ and PaO_2_
Quantity of tissue perfusion, per volume/weight
Quality of tissue perfusion, e.g., depending on tissue type, functional capillary density, flow pulsatility, velocity, rheology [39]
O_2_-offload ability of blood, depending on factors affecting P_50_ (pH, PCO_2_, 2,3-DPG concentration, temperature, or the type of hemoglobin) [39]
Oxygen consumption, e.g., depending on the metabolic state of cells
Outflow hemodynamics, e.g., post-capillary venous stasis

## Monitoring of systemic oxygenation

Clinical signs of tissue hypoperfusion and oxygenation include cyanosis, mottled skin coloration and a prolonged capillary refill time after fingernail compression. However, these signs may be absent under certain circumstances (e.g., cyanosis may be absent in anemic patients despite marked hypoxia), require experience in assessment and are less suited for objective assessment and quantitative documentation, making a follow up of these findings difficult. In addition, sensitivity and specificity of these clinical signs remains controversial, raising the need for advanced technologies to measure systemic and tissue oxygenation.

### Partial pressure of oxygen in blood and gas samples

Oxygen tension in a blood sample measured during blood gas analysis is determined by a polarographic electrode. These electrodes are often referred to as “Clark” electrodes, named after its inventor Clark [9]. A Clark electrode basically consists of a platinum cathode and silver/silver chloride anode in an electrolyte solution and is separated from the blood sample by a membrane which is permeable to gases but not to ions or contaminants of the sample. As a voltage is applied at the platinum electrode (usually −0.6 V with respect to the anode), oxygen molecules diffusing through the membrane are reduced at the cathode:

$$ {\text{O}}_{2} + 4{\text{e}}^{ - } + 2{\text{H}}_{ 2} {\text{O}} \to 4{\text{OH}}^{ - } $$

and silver is simultaneously oxidized at the anode:

$$ 4{\text{Ag}} \to 4{\text{Ag}}^{ + } + 4{\text{e}}^{ - } \quad {\text{and}}\quad 4{\text{Ag}}^{ + } + 4{\text{Cl}}^{ - } \to 4{\text{AgCl}} $$

The resulting flow of electrons generates a current. Since the flux of oxygen through the membrane is directly proportional to its partial pressure in the sample, the current is a linear function of blood pO_2_ [10].

A similar electrode, the fuel cell, is commonly used to analyze oxygen partial pressure in a gas [11]. The fuel cell has a gold cathode and a lead anode, and in contrast to the Clark-electrode, no external voltage is required because the cell itself acts as a battery. As any other battery, the fuel cell is eventually exhausted and needs to be replaced regularly. Just as with the Clark-electrode, oxygen is reduced at the cathode and the generated current is proportional to oxygen tension. Traditionally, the usefulness of fuel cells was limited by a long response time of about 15–30 s. Newer generation models offer fast response times making the technology suitable for breath-to-breath analysis of in- and expiratory oxygen concentrations (e.g., fast O_2_ sensor, used in Julian-, Cato-, and Cicero anesthesia machines, Dräger, Lübeck, Germany). Oxygen concentrations can be determined because oxygen partial pressure and oxygen concentration are directly proportional with the barometric pressure being the coefficient of proportionality. Therefore, to determine oxygen concentration, barometric pressure is either determined simultaneously or the fuel cell is calibrated at a given barometric pressure with a gas of known oxygen concentration.

Another common technique of measuring oxygen partial pressure in a gas makes use of the paramagnetic properties of oxygen. A first paramagnetic oxygen analyzer was described by Pauling et al. [12]. In contrast to most other gases used in anesthesia and intensive care medicine, oxygen is attracted into a magnetic field and this causes measurable changes in certain parameters such as gas pressure or thermal conductance. For example, when a sample gas is passed through a magnetic field, attraction of oxygen molecules by the magnetic field results in a drop of pressure in the upstream volume of gas if the flow of gas is restricted. Modern pressure sensitive paramagnetic analyzers use two restricted streams of gas, the sample gas and a reference gas, which are conducted through a pulsed electromagnetic field. Upstream to the magnet, both gas flows are connected via a pressure transducer diaphragm, and when the electromagnetic field is active, the pressure will drop more in the gas containing more oxygen than the other gas, resulting in a displacement of the diaphragm towards the gas with the higher oxygen concentration [13]. Since the electromagnetic field is pulsed, i.e., turned on and off with a high frequency, the pressure transducer diaphragm oscillates. The amplitude of oscillations is proportional to the oxygen partial pressure, and changes in amplitude reflect real-time changes of oxygen partial pressure, allowing continuous breath-to-breath analysis of oxygen concentrations.

A new generation of paramagnetic analyzers makes use of the fact that thermal conductance of oxygen is altered by a magnetic field. The gas sample is heated to a constant temperature and a pulsed electromagnetic field causes small fluctuations in conductance and hence in temperature, and these measured fluctuations are proportional to oxygen partial pressure. This new technology has been incorporated in the Zeus anesthesia machine (Dräger, Lübeck, Germany).

### Oxygen saturation

Different notations and definitions for the term “saturation” exist and medical literature is not consistent on their use. In line with prevalent clinical practice, we use the term oxygen saturation as generic term, and distinguish between “functional” and “fractional” saturation. Since the term “saturation” in its original sense implies that the system can be fully saturated, it is principally reserved to denote oxygenated hemoglobin as a fraction or percentage of total active hemoglobin, i.e., total hemoglobin that can potentially bind oxygen (oxyhemoglobin + deoxyhemoglobin) [14]. Clinically, this is often referred to as “functional” saturation, and is abbreviated as SO_2_. In contrast, “fractional” saturation is the fraction or percentage (then often abbreviated as HbO_2_ [%]) oxyhemoglobin of total hemoglobin, i.e., the sum of oxyhemoglobin, deoxyhemoglobin, COHb and MetHb, and is therefore not a “saturation” in its original sense.

Spectrophotometric methods are commonly used to determine SO_2_. These methods are based on the fact that oxygenated and deoxygenated hemoglobin possess different optical properties, and can be used in vivo and in vitro. Oxygenated hemoglobin absorbs less light at the red wavelength spectrum than deoxygenated hemoglobin. The opposite is true at the infrared spectrum.

In vivo, pulse oximetry uses spectrophotometry to determine S_p_O_2_ depending on pulsatile changes in blood volume in the optical pathway. Common clinical notation uses the subscript “p” to denote that saturation is measured by pulse oximetry. The concept was devised by Aoyagi [15]. Light at (usually) two wavelengths, typically 660 (red) and 940 nm (infrared), is transmitted through tissue to a photocell [16–18]. This tissue is often a finger or earlobe, however also forehead surface pulse oximeters are available which detect light that travels laterally between the emitters and the photocell. The photocell cannot distinguish whether the light originates from the red-, infrared-, or ambient light. Hence, the emitters are alternately switched on and off, up to several hundred times per minute, so that the photocell signal can be matched to either light emitting diode. Intermittently, both diodes are switched off to allow the detection and correction of ambient light interference [18].

Absorption of red- and infrared light detected by the photocell consists of background absorption by tissues, venous and capillary blood as well as by non-pulsatile arterial blood. In addition, there is a pulsatile component of light absorption due to changes in volume attributable to arterial blood. The magnitude of pulsatility is the same for both wavelengths, and the pulse oximeter calculates the ratio of the pulsatile component and the baseline component of light absorption for each wavelength. In turn, the ratio of the two ratios is calculated, which is directly related to SpO_2_ [18].

Although pulse oximetry is a non-invasive and continuous standard-of-care, it also has some drawbacks. First, a pulsatile flow in a peripheral tissue such as a finger is required, therefore the technology is not suitable during non-pulsatile flow (cardiopulmonary bypass), may be unreliable when peripheral tissues are not adequately perfused, e.g., in shock states or during hypothermia, and is subject to motion artifacts, e.g., during patient transport [19, 20]. Second, pulse oximetry has limited accuracy when SO_2_ is below about 80 % [21]. This, however, is clinically of minor importance as any calculated S_p_O_2_ below 80 %, even with limited accuracy, indicates severe hypoxemia and requires immediate action. Third, by using only two wavelengths, pulse oximeters cannot account for the influence of dyshemoglobinemia on oxygen saturation [22]. Carboxyhemoglobin (COHb) shows a similar light absorption at 660 nm as oxyhemoglobin and therefore causes erroneously high S_p_O_2_ readings in patients with carbon monoxide intoxication. Methemoglobin (MetHb) has a similar light absorption at 660 and 940 nm and at high concentrations tends to bias S_p_O_2_ towards a reading of 85 %.

Reflectance spectrophotometry is another, yet similar technique to monitor systemic oxygenation and perfusion in vivo. In contrast to pulse oximetry, this technique allows continuous assessment of intravascular oxygen saturations, however it is invasive and requires insertion of an intravascular catheter into the vessel of interest. Light of different wavelengths is transmitted through a fiberoptic filament incorporated in the catheter, and reflected light is transmitted back through another filament to a photodetector [23]. Clinically, this technique is most often incorporated in a pulmonary artery catheter for continuous monitoring of mixed venous oxygen saturation, which will be discussed in more detail later. Recently, a probe has become available which can be placed via a standard central line, allowing for routine continuous clinical assessment of central venous saturation (CeVox, Pulsion Medical Systems, Munich, Germany).

In vitro, older blood gas analysis devices used to estimate SO_2_ from pO_2_, however such calculated values do not account for shifts in the oxygen-hemoglobin-dissociation curve and may therefore be misleading in the clinical situation. Hence, SO_2_ is usually measured rather than calculated in modern blood gas analyzers by using the same spectrophotometric methods as outlined above. Such devices use co-oximetry with additional wavelengths to determine COHb and MetHb concentrations [24]. Hence, co-oximeters are capable of also reporting fractional saturation:

$$ {\text{HbO}}_{2} [\% ] = 100 \times \frac{\text{oxyhemoglobin}}{{{\text{oxyhemoglobin}} + {\text{deoxyhemoglobin}} + {\text{COHb}} + {\text{MetHb}}}} $$

Depending on the type of blood sample drawn, this technique can intermittently monitor arterial, capillary, central venous or mixed venous saturations, each of which may allow conclusions on systemic oxygenation.

### Systemic oxygen delivery

As stated earlier, systemic oxygen delivery is the product of arterial oxygen content (C_a_O_2_) and cardiac output (CO). Therefore, while we have previously discussed tools necessary to calculate C_a_O_2_, measurement of CO is also required for a comprehensive assessment of oxygen delivery.

The clinical gold-standard of CO measurement is the thermodilution method using a pulmonary artery catheter (PAC) [25]. Herein, a bolus of cold fluid (e.g., at room temperature) is injected through the central venous port of the PAC, and changes in temperature are measured downstream by a thermistor in proximity to the catheter tip in the pulmonary artery. Cardiac output is inversely proportional to the area under the temperature/time curve, and can be calculated by the modified Stewart-Hamilton equation. This equation also considers the volume and temperature of the injectate, blood temperature, specific gravities and specific heat of injectate and blood, as well as a correction constant depending on the type of catheter used [26].

Limitations of the technique include inevitable variations of volume and speed of cold bolus injection and variations due to bolus injection at different time points of the respiratory cycle. This can result in variations of up to 25 % between two subsequent measurements, and therefore averaging a minimum of three bolus measurements is generally recommended [27]. Other errors, e.g., due to heat transfer from surrounding tissue or warming blankets, due to intracardiac shunts or tricuspid regurgitation, or due to malplacement or migration of the PAC, may lead to systematic bias.

Pulse contour analysis is a less invasive alternative for CO determination and does not require insertion of a PAC [28]. Stroke volume is proportional to the area under the systolic portion of the arterial pressure curve and to the pulse pressure, i.e., the difference between systolic and diastolic pressure. Mathematical algorithms are used to deduce CO from the arterial pressure curve. However, since the arterial pressure curve also depends on compliance and resistance of the arterial vasculature, prior individual calibration is usually required. Transpulmonary indicator-dilution techniques are often used for calibration, which commonly use a cold fluid bolus (PiCCO, Pulsion Medical, Munich, Germany) or lithium (LiDCO, LiDCO ltd., London, UK) as indicator [29, 30]. In analogy to the PAC thermodilution technique, CO is inversely proportional to the area under the concentration/time curve, however with the difference that concentration (or temperature in the case of PiCCO) is not measured in the pulmonary artery but in a systemic artery. Another commercially available pulse contour analysis device, the FloTrac/Vigileo system (Edwards Lifesiences, Irvine, CA, USA), requires no calibration but estimates arterial compliance and resistance based on a mathematical algorithm based on patient’s age, sex and body surface area [31].

Echocardiographic based calculations of CO are even less invasive than pulse contour analysis as they do not require disruption of tissue barriers by insertion of venous or arterial catheters. Methods include calculation of stroke volume and CO by determining the difference between enddiastolic and endsystolic ventricular volume, or combine measurements of transvalvular blood flow velocity with determination of the respective valve area.

Measurement of aortic blood flow velocity using an esophageal Doppler probe offers a relatively simple, quick and continuous alternative which can be used for bedside monitoring of CO [32, 33]. Basically, ultrasound emitted by a transducer towards the descending aorta is subject to a change in its frequency when reflected by moving erythrocytes (Doppler principle). This frequency shift is directly proportional to blood velocity, allowing to construct a velocity–time profile of aortic blood flow. The area under the velocity–time curve, in turn, is proportional to stroke volume, which can be estimated by an algorithm based on the patient’s age, height and weight (CardioQ, Deltex Medical, Chichester, UK).

Other less commonly used non-invasive techniques for estimation of CO include partial carbon dioxide rebreathing techniques based on the Fick principle and thoracic electric bioimpedance.

### Downstream markers of systemic oxygenation

Markers of arterial oxygenation as assessed by p_a_O_2_, S_a_O_2_, C_a_O_2_ and DO_2_ as described in the previous paragraphs can be considered “upstream” markers of systemic oxygenation as they describe systemic oxygen supply to the organs. Other markers, measured “downstream” the tissues in venous blood can also give valuable indirect information on tissue metabolism and oxygenation. One of these parameters is mixed venous oxygen saturation (S_v_O_2_), which can either be measured by blood gas analysis in vitro or by reflectance spectrophotometry via a PAC in vivo as detailed above. Under physiologic conditions, about 25 % of total delivered oxygen is consumed by the organs [7, 8]. When oxygen delivery decreases, this is initially compensated by higher oxygen extraction, which results in a decrease in S_v_O_2_. On the other hand, marked increases in oxygen delivery, for example due to hyperdynamic circulation, may be associated with an increased S_v_O_2_. Moreover, decreases in tissue oxygen consumption, e.g., due to hypothermia or loss of viable tissue, are also associated with increased S_v_O_2_. Hence, since mixed venous blood comprises blood from all organs, S_v_O_2_ reflects the global balance between systemic O_2_ delivery and O_2_ consumption. Similarly, the difference between arterial and mixed venous oxygen content (AVDO_2_ = C_a_O_2_–C_v_O_2_) is directly proportional to oxygen consumption (VO_2_) and inversely proportional to cardiac output (CO) as described by the Fick principle:

$$ {\text{AVDO}}_{2} = \frac{{{\text{VO}}_{2} }}{\text{CO}} $$

Hence, increases in AVDO_2_ often reflect a decrease in cardiac output unless oxygen consumption is markedly increased. Vice versa, the principle can be used to determine oxygen consumption in patients monitored with PAC, in whom CO can be determined by thermodilution.

Central venous saturation (S_cv_O_2_) is often used in clinical practice as a surrogate for mixed venous saturation with the advantage that a central venous access without instrumentation of the pulmonary artery is sufficient. Differences between central and mixed venous saturations arise from the fact that blood from the superior caval vein has a lower physiologic saturation as from the inferior caval vein [34]. This relationship, however, may be reversed in shock due to a relative high oxygen extraction by splanchnic organs while cerebral perfusion and hence oxygen extraction in the brain remains well preserved [35]. Admixture of blood from the coronary sinus in the right atrium also contributes significantly to differences between central and mixed venous saturations as the heart receives about 5 % of cardiac output and has a high oxygen extraction rate compared to other organs. Despite these limitations, several investigators have reported a high correlation (≥0.88) between mixed and central venous saturations in different patient populations [36, 37], however some studies report a poorer correlation for patients in shock [35, 38, 39]. The findings suggest that S_cv_O_2_ can principally be used as a surrogate for S_v_O_2_, however, especially in patients with shock the results should be interpreted with care and in the context of other measured parameters. Nonetheless, low values are alarming and may be clinically relevant, and S_cv_O_2_ is frequently used as a therapeutic target to improve oxygen delivery in critically ill patients. In the landmark study on early goal directed therapy of sepsis by Rivers et al. [40], one of the therapeutic goals was to maintain the S_cv_O_2_ above 70 %, and patients randomized to the goal directed therapy group showed a lower in hospital mortality than patients assigned to standard therapy (30.5 vs 46.5 %). Note that obtaining a sample of central venous blood requires a proper position of the central venous catheter on the transition from the superior vena cava to the right atrium, and therefore catheter position should be confirmed prior to using S_cv_O_2_ as diagnostic tool or therapeutic goal.

While decreases in oxygen delivery can initially be compensated by increased oxygen extraction, this compensation is insufficient to maintain oxidative metabolism when oxygen delivery drops below a critical threshold [41]. Oxygen consumption becomes DO_2_ dependent and lactate is produced as the end product of anaerobic glucose metabolism [42]. Hence, lactate concentrations are a second downstream marker, which may indicate tissue hypoperfusion and hypoxia. This however is not specific for inadequate systemic oxygen delivery because hyperlactatemia may also be due to regional ischemia, due to mitochondrial dysfunction in septic patients or due to increased aerobic glycolysis when pyruvate production exceeds pyruvate dehydrogenase capacity, such as in conditions associated with increased circulating catecholamine levels or cytokine release [43].

## Monitoring of tissue oxygenation

On the systemic level, systemic blood flow (cardiac output, CO) and oxygen delivery (DO_2_) are mathematically linked by simple formula, i.e., DO_2_ = CO × CaO_2_. However, on the regional level, the link between tissue perfusion and oxygenation is more complex. Although total perfusion into a microcirculatory area may be normal, perfusion abnormalities within this microcirculatory area (e.g., by shunting, recently reviewed by De Bakker et al. [44]) may still lead to local tissue hypoxygenation. Therefore the following paragraphs focus on the assessment of tissue oxygenation, not flow.

Several techniques for regional monitoring of tissue oxygenation have been developed over the last decades, however, only within the last years there is growing effort to implement those techniques into the clinical practice. The reasons for this are basically twofold: On the one hand, there is an increasing acceptance that regional parameters add significant value not only to research but also to clinical decision making and outcome. On the other hand, traditional techniques to measure regional oxygenation were not suitable for broad clinical use and only current developments appear to open this clinical window.

With some exception, techniques to measure tissue oxygenation reflect the balance between regional oxygen delivery to the tissue, oxygen uptake (or: consumption in the steady state) and the state of venous tissue drainage. In pathologic states, frequently more than one factor is altered affecting regional tissue oxygenation. On the delivery site tissue oxygenation depends on hemoglobin concentration, SO_2_ and PO_2_, combining to the local blood’s O_2_ content. In contrast to in vitro use (Lex-O_2_-Con, measuring blood O_2_-content by a galvano-coulombmetric method, as applied by others [45] and us [46]), no broadly applicable technique is available to determine in vivo blood O_2_ content. On the transit from the inflow site to the venous part of the microcirculation, SO_2_ and PO_2_ of the blood are decreasing, depending on the grade of shunting and capillary oxygen extraction. Herein, technologies are available to measure both, microvascular PO_2_ or microvascular SO_2_ [47]. Differences exist between the macro- and the microcirculation, e.g., the hemoglobin concentration within the microcirculation may be markedly lower than in the systemic circulation. In addition, the relation between PO_2_ and SO_2_, i.e., the oxygen dissociation curve, may be right-shifted in the micro- versus the macro-circulation, e.g., because of a lower pH and a higher PCO_2_. These differences have to be taken into account when interpreting PO_2_ and SO_2_ data of tissue oxygenation.

### Measurement of PO_2_ in the microvasculature and tissue

#### Palladium porphyrine phosphorescence-based methods

These are optical methods to measure (microvascular) tissue oxygenation, suitable for in vivo application. This technique exploits the fact that, after pulsed light stimulation, the phosphorescence quenching of certain palladium containing porphyrine compounds (Pd-*meso*-tetra (4-carboxyphenyl) porphine; Porphyrin Products, Logan, UT) depends on the PO_2_ of the surrounding milieu. To allow for intravascular measurements, the palladium porphyrine compound is coupled prior to injection to human albumin, forming a stable high-molecular-weight complex to stay within the vascular compartment during the measurement period. After application of the dye, one or more probes are positioned above tissues of interest, e.g., of the gastrointestinal tract, the kidneys or the brain [48]. Since signal intensity increases with a drop in PO_2_, this technique is suited for measurement of low PO_2_ values. This technique allowed to gather important insights into microcirculatory tissue oxygenation in multiple pathologic conditions, including sepsis models [49], hemorrhage or hemodilution [48]. Furthermore, established or experimental treatment modalities have been (re-)evaluated applying this technique [47, 50]. Disadvantages of this technique relate to the required palladium dye, particularly that intravascular confinement decrease over longer periods and, regarding patient use, that the palladium dye is regarded toxic for human use.

However, the described principle of oxygen pressure measurement has been embedded into catheter type probes and may be further developed to needle type applications [51] also for patient use.

#### Oxygen pressure electrodes

Like palladium porphyrine phosphorescence techniques, also oxygen electrodes measure tissue PO_2_, rather than SO_2_. While palladium porphyrine phosphorescence quenching is applied to measure predominantly intravascular PO_2_, assuming intravascular confinement of the tracer dye, PO_2_ electrodes do not discriminate between intra- or extravascular PO_2_ and thus measure a composite PO_2_. The basic principle of PO_2_ electrodes is described above. Oxygen electrodes of various types have been developed for multiple indications, ranging from transcutaneous application to conjunctival PO_2_ measurement. In the field of critical care, PO_2_ electrodes have been established to measure tissue oxygenation in several animal models [52]. Herein, superficial tissue PO_2_ can be measured by Clark-type multichannel/multi-wire surface electrodes, usually after calibration (e.g., N_2_ and room air). One electrode may consist of several platinum wires, each of which has a diameter of 15 μm, representing an individual measuring point and one Ag–AgCl reference electrode. These electrodes are reported to measure oxygen tension of the superficial epithelial layers [53]. Thus, although surface PO_2_ electrodes have a restricted measurement depth this technique allows determination of tissue PO_2_ without tissue damage and interference with the microcirculation [54]. Measurement at different locations allows to address heterogeneity of tissue oxygenation; the obtained values of tissue oxygenation are the net result of nutritive blood flow and tissue oxygen consumption [55]. Limitation of this technique is the restricted penetration depth (surface electrodes); if deeper measurements are required, PO_2_ needle electrodes are available. However, these are traumatic and even microscopic bleedings affect tissue PO_2_. A further methodological limitation is, that PO_2_ electrodes do not resemble the microvascular PO_2_, but are biased towards the arterial component, because they are largely affected by the higher PO_2_ values within the catchment volume. Thus, although PO_2_ electrodes are generally established to measure blood PO_2_, these limitations prevent a widespread use of PO_2_ electrodes to measure tissue PO_2_.

#### Measurements of microvascular oxygen saturation

Closely related to the PO_2_ is the resultant oxygen saturation of oxygen binding molecules, prototypically hemoglobin. The two most broadly applied techniques to measure regional oxygen saturation are near infrared spectroscopy (NIRS) and reflectance spectroscopy. Given the rapidly growing interest in NIRS in multiple fields of medicine in the last decade, an entire chapter in this issue is dedicated to this technique and description is therefore omitted here. The second promising technique which made the transition from an experimental method to a technique that is suitable for clinical use is white light spectroscopy and is detailed here.

Spectroscopy using white light is used to measure hemoglobin oxygen saturation within the microcirculation (μHbO_2_). Depending on reference, the technology is termed reflectance spectroscopy, remission spectroscopy or spectrophotometry. A light source emits white light with a wavelengths of 500–800 nm which, via fiber glass and a specific probe is introduced into the tissue of interest. Within the tissue, the light is scattered, e.g., at mitochondria and therefore spreads diffusely. Only a minor portion of the photons eventually returns back to the surface of the tissue. Between the emitter and the detector, both placed on the surface of the tissue with a defined distance, a banana or boomerang shaped light arc can be imagined, resembling the predominant way of photons between emitter and detector. In case of a narrow distance between emitter and detector, this light arc will be small and therefore superficial, in case of a larger emitter/detector distance, the light arc will be larger and reach deeper into the tissue. This principle has been used to develop probes allowing depth selective measurements of tissue oxygenation. The range of current probes allows superficial measurements of 100 μm to measurement depth of 16 mm, e.g., for deeper muscle measurement.

Besides flat probes to be placed (or sutured) on the tissue of interest, small probes with emitter and detector integrated at the tip of a thin light guide probe are available. The dimension (e.g., diameter 2 mm) and flexibility of this miniaturized probes allow an endoscopic application, and have been applied by us in animal studies [56, 57], as well as in volunteers [58] and patients [59, 60].

On its way from emitter to detector though the tissue, a part of the light spectrum is absorbed when the white light interacts with hemoglobin. Depending on the oxygenation status of hemoglobin, the light will partly be absorbed and changing its spectrum. From the resultant spectrum, a software fed with spectra ranging from completely des-oxygenated to fully oxygenated hemoglobin calculated the hemoglobin oxygenation within the tissue. In modern devices, the spectrum is analyzed in a wavelength range between 500 and 810 nm and circa 300 wavelengths are analyzed simultaneously. Modern systems allow a continuous measurement of tissue oxygenation, allowing generation of oxygenation histograms or tracking rapid oxygenation changes over time on-line.

The principal methodology has been established for decades, however, the original generations of measurement systems (e.g., the EMPHO II, Bodensee Geratetechnik, Uberlingen, Germany) were heavy men-high boxes, unsuited for bed-side use. Intermediate device generations (e.g., AbTisSpec, LEA, Germany) or current device generations (O2C, LEA, Germany) are suitcase format, thus applicable for patient use. Miniaturization is based on replacement of original bulky components, e.g., an original motor-driven rotating filter wheel mechanism has been replaced by a CCD-spectrometer and the bulky xenon high-pressure lamp by a smaller white light source [61].

As described, the spectrophotometric μHbO_2_ signal predominantly derives from the microvasculature—and not the larger vessels. This is explained by two factors, together causing that full blood (and hematoma) tend to completely extinct the white light introduced into the tissue: The absence of mitochondria in erythrocytes limits scattering of light and thus permits deeper penetration of light into the vessel, where it is finally totally absorbed and thus lost for photometric analysis.

Absorption is further enhanced by the large amount of hemoglobin in major vessels (and hematoma), with hemoglobin being the largest light absorber in normal tissue. The notion of a diminished (or lost) signal by light absorption due to contact with hemoglobin has been exploited to derive a measure of the regional amount of hemoglobin (rHb) in the catchment volume. This measurement is reported to represent the hemoglobin concentration per tissue volume, not necessarily per blood volume.

Manufacturers of tissue oxygen monitors tend to promote cut off or intervention levels, probably to underline the ease of use of those techniques. In the case of spectrophotometry the manufacturer reports a critical SO_2_ cut off of (only) 10 %. These cut off values are probably only valid for certain tissue types. A general critical SO_2_ is unlikely, thus critical SO_2_ values will have to be defined for various tissues separately. Given an arterial SO_2_ of almost 100 % and a central venous SvO_2_ of 75 %, one may at first glance expect tissue SO_2_ to be 85–90 %. However, since the SO_2_ signal derives from the microcirculation, distribution of blood within the microcirculation is a crucial factor: Although the capillaries have the largest total cross sectional area of all vascular compartments (e.g., supporting O_2_ delivery to the tissue), the largest blood volume is present within the venous site of the circulation. Thus the majority of blood within the (micro-)circulation is in the post-extraction section (about 70–85 %) and therefore the overall signal will predominantly reflect the venous side of the microcirculation [61]. By this, this technique allows the measurement of oxygen saturation of hemoglobin (μHbO_2_) at the *venous end of the capillaries*, referred to as the “last meadow” of the circulation. Thus, one has to consider that the SO_2_ values obtained are post-extraction (post-capillary, venular) SO_2_-values, and may therefore be expected considerably lower than the arithmetic between arterial and central venous blood.

Besides the mentioned applications in anesthesia and critical care reflection spectroscopy has been applied to monitor surgical results after tissue flap transplantations, or monitoring of wound healing [62].

## Cellular oxygenation

The microcirculation can be regarded as a highly active interface between the systemic circulation and the cells. Many disease processes, e.g., sepsis, can be better understood (and thus treated) when the role of the microcirculation within the disease is clarified. However, ultimately, the microcirculation serves to supply the single cell with nutrients, most critically oxygen, and to return metabolic (by-)products to the venous system. Therefore, monitoring the oxygenation of the cells including the mitochondria (as actual oxygen consumer) appears an attractive next step in this context. In recent publications, Mik et al. [63] were able to demonstrate the feasibility to measure mitochondrial PO_2_ and to address a number of important questions regarding mitochondrial oxygenation. This technique is based on the oxygen-dependent quenching of the delayed fluorescence of protoporphyrine IX. Since this protoporphyrine compound is an endogenous product of metabolism, future application of this technique also in humans appears possible [64].

## Future perspectives to measure tissue oxygenation

Besides the trend to develop more compact devices, allowing routine bed side monitoring of patients, a future trend is the combined assessment of different measurement techniques in one device, ideally one measurement probe. Since perfusion and oxygenation are not interchangeable, combined measurement of flow and oxygenation may assist to discriminate the origin of impaired tissue oxygenation. A low oxygenation may result from low inflow, high extraction (consumption) or restricted venous outflow. An example for a combined monitoring device is the O_2_C monitor (LEA, Germany), combining spectrophotometry and laser Doppler based perfusion measurement [65]. In turn, videomicroscopic techniques like OPS- or SDF-based perfusion imaging [66] could be combined with oxygenation measurements, possibly allowing oxygen distribution measurement within the microcirculation. The combined efforts of engineers in the field, basic researchers and ultimately clinicians will hopefully lead to improved diagnostics of tissue dysoxia, defined as condition where oxygen availability limits metabolism, and ultimately improved therapy and outcome.
